# Update on Idiopathic Intracranial Hypertension Management

**DOI:** 10.1590/0004-282X-ANP-2022-S110

**Published:** 2022-08-12

**Authors:** Marcio Nattan Portes Souza, Barbara de Alencar Leite Costa, Felipe Reinaldo Deus Ramos Santos, Ida Fortini

**Affiliations:** 1 Universidade de São Paulo, Faculdade de Medicina, Hospital das Clínicas, São Paulo SP, Brazil.

**Keywords:** Pseudotumor Cerebri, Acetazolamide, Optic Nerve, Cerebrospinal Fluid, Bariatric Surgery., Pseudotumor Cerebral, Acetazolamida, Nervo Óptico, Líquido Cefalorraquidiano, Cirurgia Bariátrica.

## Abstract

**Background:**

Idiopathic Intracranial Hypertension (IIH) is a secondary headache with a steadily growing incidence. Currently, there is little evidence to guide the treatment of IIH.

**Objective:**

To review the pathophysiology of IIH, with focus on the role of obesity as a risk factor, and the implications for new therapeutic perspectives.

**Methods:**

in this narrative review, we summarized the current knowledge on treatment options highlighting available evidence for managing intracranial hypertension, obesity, and headache.

**Results:**

Clinical Presentation: headache is the most common symptom and a significant cause of quality-of-life impairment. Visual loss is common in the diagnosis. Pathophysiology: there is no unified theory able to explain all symptoms and the evolution of the disease. There is growing data pointing to metabolic changes and obesity with a central role in IIH pathophysiology. Treatment: most published data on IIH treatment is related to pressure control and protection from visual loss. Acetazolamide and cerebrospinal fluid diversion are the best options available. Optic nerve sheath fenestration might be useful to temporally control the pressure over the optic nerve and thus protect from visual deterioration. Recently, venous sinus stenting has proven to be a safe option in selected cases. Finally, bariatric surgery has proven to effectively control elevated intracranial pressure.

**Conclusion:**

IIH is a potential cause of high disability. Early recognition is important, and treatment should be tailored to the needs of each case. There is a lack of research on headache management, which might persist after ICP control.

## RESUMO

**Antecedentes:**

A Hipertensão Intracraniana Idiopática (HII) é uma cefaleia secundária com incidência crescente. Atualmente há pouca evidência disponível na literatura referente ao manejo da HII.

**Objetivo:**

O entendimento da fisiopatologia e o papel central da obesidade como fator de risco para HII abriu novas perspectivas de tratamento.

**Métodos:**

nessa revisão narrativa, objetivamos revisar as principais opções de tratamento disponíveis atualmente para o manejo da HII, controle da obesidade e da cefaleia.

**Resultados:**

Apresentação clínica: a cefaleia é o sintoma mais comum e uma importante causa de impacto na qualidade de vida, e o déficit visual é um achado comum no diagnóstico. Fisiopatologia: atualmente não existe uma teoria unificada capaz de explicar satisfatoriamente os sintomas e a evolução da doença. Um crescente corpo de evidências aponta para o papel central das alterações metabólicas e da obesidade na fisiopatologia da HII. Tratamento: a maioria dos dados publicados sobre HII estão relacionados a medidas para controle da hipertensão intracraniana e proteção da visão. Acetazolamida e Derivação Ventriculo Peritoneal são as principais alternativas com esse fim. A fenestração do nervo óptico pode ser útil como medida termporaria de controle da pressão sobre o nervo óptico e proteção contra a progressão do déficit visual. Cirurgia bariátrica se mostrou efetiva no controle da pressão intracraniana.

**Conclusão:**

A HII é uma causa importante de incapacidade cujo reconhecimento precoce é importante. O tratamento deve ser individualizado. Atualmente há uma importante defasagem de evidências sobre o manejo da cefaleia nesse grupo de pacientes.

## INTRODUCTION

Idiopathic intracranial hypertension (IIH) is a secondary headache caused by an elevation of the intracranial pressure (ICP). IIH typically affects obese women of childbearing age^1^. In a recent study in the UK, the estimated annual incidence was 4.7, which represents a growth of 108% in 14 years, and parallels the growth of obesity prevalence^2^. Healthcare resource utilization has also grown as reflected by a rise of 442% in hospital admissions in 12 years^2^.

## CLINICAL PRESENTATION AND DIAGNOSIS

Headache is the most common symptom, frequently accompanied by transient visual obscurations, pulsatile tinnitus, back pain, dizziness, neck pain, visual loss, cognitive disturbances, radicular pain, and horizontal diplopia^3^. Papilledema is found in the vast majority of patients with confirmed IIH^4^, and the grade of papilledema is directly related to the risk of permanent visual loss and treatment failure^5^. At diagnosis, visual impairment is present in over 80% of patients, and some degree of permanent visual loss is observed in 10% of patients^6^. Another less common IIH symptom is diplopia, which is due to sixth nerve palsy, usually related to a more severe presentation. 

The current diagnostic criteria for IIH requires the presence of papilledema, neuroimaging without evidence of a secondary cause of intracranial hypertension, normal CSF composition, and elevated lumbar pressure^1^. The detailed diagnosis criteria is found in Table 1. IIH rarely presents without papilledema. In these cases, the alternative criteria require the presence of 3 of the 4 typical indirect findings of intracranial hypertension: empty sella, flattering of the posterior aspect of the globe, distention of the perioptic subarachnoid space with or without a tortuous optic nerve, and transverse venous sinus stenosis in the neuroimaging.

**Box 1.** Modified diagnostic criteria for idiopathic intracranial hypertension.

A. Papilledema
B. Normal neurologic examination except for cranial nerve abnormalities
C. Neuroimaging: Normal brain parenchyma without evidence of hydrocephalus, mass, or structural lesion and no abnormal meningeal enhancement on MRI, with and without gadolinium, for typical patients (female and obese), and MRI, with and without gadolinium, and magnetic resonance venography for others; if MRI is unavailable or contraindicated, contrast-enhanced CT may be used
D. Normal CSF composition
E. Elevated lumbar puncture opening pressure (≥250 cmH2O CSF in adults and ≥280 cm H20 CSF in children) in a properly performed lumbar puncture

## PATHOPHYSIOLOGY

The pathogenesis of IIH is not yet clear. Dysfunction in the balance between CSF secretion and drainage seems to be the underlying cause. The high prevalence of obesity among IIH patients points to the role of metabolic changes, but this association is complex as obesity is a common condition and IIH is a rare disease. Furthermore, weight loss leads to clinical improvement^7^, and weight gain is linked to IIH recurrence^8^. Recent evidence supports the role of adipokine, leptin, Glucagon-like peptide-1, and 11ß-hydroxysteroid dehydrogenase (11-ßHSD1) in the pathophysiology of IIH, highlighting the metabolic nature of this condition^9^. Understanding of the metabolic pathways involved in ICP regulation has led to the development of novel targeted therapies such as bariatric surgery^10^, 11-ßHSD1 inhibitor^11^ and GLP-1 receptor agonist.

In IIH patients, the elevated cranial pressure induces microstructural compression of the optic nerve, impairing axoplasmic flow and causing papilledema and visual loss^12^. The main goal of therapies directly targeting ICP control is to reduce transient and permanent visual loss.

Headache is the most common symptom and the cause of significant quality of life impairment for IIH patients^13^. The most common phenotype is a migraine-like headache^14^. Although headache seems to be related to the raised ICP, a substantial number of patients present with a persistent headache after ICP normalization^15^. Despite the high frequency and burden of headache for IIH patients, to date little is known about the pathophysiology, and no directed trials have investigated headache treatment.

## TREATMENT

IIH treatment has three main objectives: body weight loss, vision protection, and headache control.

To date, weight loss is the only modifying disease measure. All patients with BMI ≥ 30 kg/ m² should be oriented for weight control. A multidisciplinary team (nutritionist, endocrinologist) is sometimes necessary to help patients in weight management. The precise amount of weight reduction that should be aimed for IIH remission is not established. However, 5-15% weight gain is a risk factor for developing IIH, so it is reasonable to advise patients to lose at least 15% weight^16^.

## PHARMACOLOGICAL TREATMENT

The main goal of the pharmacological treatment of IIH is to protect from visual loss. Acetazolamide is the first-choice drug, and its use in patients with mild visual loss can result in improvement in visual field function and quality of life^17^. The starting dose is 250-500mg twice a day, and it can be titrated until a maximal dose of 4g daily. Common adverse effects are diarrhea, dysgeusia, fatigue, nausea, paresthesia, tinnitus, vomiting, depression, and rarely renal stones. Periodic monitoring of serum electrolytes and venous gasometry should be performed^18^.

An open-label study suggested similar efficacy of acetazolamide and topiramate (50-200mg daily)^19^. Topiramate might be very helpful considering the frequency of obesity and migraine among IIH patients. Furosemide (maximal dose of 40mg twice a day) is a third option if the previous drugs cannot be tolerated^20^.

## SURGICAL PROCEDURES

In the presence of imminent visual deterioration, surgical management is an effective alternative for visual protection. CSF diversion and optic nerve sheath fenestration (ONSF) have been employed in the short term. 

## CSF DIVERSION SURGERY

In many centers, the neurosurgical CSF diversion is a surgical first-line choice. Ventriculoperitoneal shunt (VPS) and lumboperitoneal shunt (LPS) are both effective in patients with progressive vision loss based on case series^18^. The shunt procedure did not reverse established visual loss but was effective in stabilizing the worsening. In 53 patients’ retrospective case series, patients who underwent a CSF shunt presented papilledema reduction and improvement in visual acuity and tinnitus. Fundus examination showed a significant reduction in the number of patients with papilledema from 92% at baseline to 65% at 6 months, 48% at 12 months, and 44% at 24 months after shunt surgery. The LPS was the most performed procedure in the studies, but with a high rate of shunt revision when compared to VPS, hence the last one is most recommended. Ideally, the surgical approach should be performed by an experienced neurosurgeon with an interest in CSF disorders^21^. 

In the case series, headache recurrence was documented in about 60-70% of patients by 1-year post-procedure. Therefore, shunts should not be indicated for the treatment of IIH-associated headaches in the absence of visual loss^22^. More than half of patients undergoing shunts will require surgical revision and about one-third underwent multiple revisions. Other complications are reported, such as abdominal pain, valve and/or circuit obstruction, infection, headaches due to low CSF pressure, subdural hematoma, and tonsillar herniation. Adjustable valves should be preferred because of the risk of low-pressure headaches. Deaths are reported in shunt placement; however, no data were obtained from IIH studies^23^. Despite the adverse events, CSF derivations are still the most available procedure in fulminant IIH or medication failure, with positive visual outcomes.

## OPTIC NERVE SHEATH FENESTRATION

The optic nerve sheath fenestration (ONSF) is a technique with a lower complication rate and no reported mortality. Therefore, ONSF is preferred by many experts when considering the re-approaches after the shunt placement^24^. The ONSF may be performed by neuro-ophthalmologists experienced with the technique, which limits access in many centers. Reports and case series have shown positive outcomes with this approach in improving visual acuity and visual field in patients with medication failure. In the majority of reported cases, ONSF was bilateral, but unilateral ONSF can be an option especially when there is asymmetric visual impairment, and may improve not only the operated eye, but also the non-operated eye. In the unilateral technique, the eye chosen for the fenestration is the one with the worst visual performance. The possibility to perform a unilateral ONSF with bilateral results reduces the time of anesthesia and possibility of complications^25^.

The rate of OSNF’s complications is low and the main adverse effects are transient, not needing further surgery procedures: double vision, anisocoria, and ocular hemorrhages. Permanent complications such as central retinal artery occlusion are reported in < 1% of the cases^26^. OSNF is also considered by some experts as a first-line approach to fulminant visual loss to protect vision while effective weight loss treatment is achieved. In case of failure, the patient should undergo a more invasive procedure such as CSF shunt, avoiding the need for multiple procedures. The results of fenestration in improving headaches are conflicting, with a single review with about 50% improvement and other studies with 20-30%. The pathophysiology of headache improvement is uncertain and may result from a placebo effect^24^. The OSNF is a safe, less invasive, and effective alternative for progressive visual loss in patients with medication failure in asymmetric papilledema causing unilateral visual loss^18^.

## VENOUS SINUS STENTING

A more recent therapeutic approach for IIH is venous sinus stenting, reported for the first time in 2002^27^. Since then, an increasing number of case series have shown promising results. In a large meta-analysis with 474 patients, the overall rate of improvement in papilledema, headache and pulsatile tinnitus was 93.7% (95% CI 90.5% to 96.9%), 79.6% (95% CI 73.3% to 85.9%) and 90.3% (95% CI 83.8% to 96.70%), respectively (28). It may be reasonable for highly selected IIH patients with venous sinus stenosis and elevated pressure gradient across the stenosis region (8mm Hg or higher) in whom standard therapies failed^18^. Regarding the procedure, venography and manometry should ideally be performed with the patient awake, along with dual or single antiplatelet drugs administered before as well as at least 3-6 months following stenting^29^.

Recurrence of IIH symptoms after stenting occurred in 9.8% (95% CI 6.7% to 13%) of patients^28^. High BMI, African-American race, female gender, pure extrinsic compression of the transverse-sigmoid junction, highly raised opening pressures and persisting papilledema post-procedure possibly increase the risk of stent failure^30^. On the other hand, patients who had higher mean pressure gradients and higher changes in pressure gradients after stent placement seem to have favorable outcomes^31^.

The rate of major complications (subdural hematoma, subarachnoid hemorrhage, thrombosis, fistulae) was 1.9% (95% CI 0.07% to 3.1%) and the overall mortality was 0%^28^. Short-lived ipsilateral, stent-adjacent headache was the most common complication (30%)^32^. The outcomes and the eligibility for the procedure were not standardized among studies, and randomized clinical trials are lacking.

## BARIATRIC SURGERY

As obesity is the main modifiable risk factor for IIH^8^ researchers have hypothesized that treatment targeting body weight control improves clinical outcome (10,14). One randomized controlled trial compared bariatric surgery (BS) (N= 33) to community weight management (CWM) (N= 33). The primary outcome evaluated was change in ICP after 12 months. Secondary outcomes included change in ICP after 24 months, visual acuity, Headache Impact Test score (HIT-6), perimetric mean deviation, and quality of life (measured by the 36-item Short Form Health Survey). At baseline, the mean BMI was 43.7 in the CWM group and 44.2 in the BS group. In the surgery arm, different procedures were accepted, and the main method was Roux-en-Y gastric bypass (44%), followed by gastric banding (37%) and laparoscopic sleeve gastrectomy (18.5%). At 12 months the mean ICP decreased from 34.8 to 26.4 cm CSF (-8.7 cm CSF; P <0.001) in the BS arm and from 34.6 cm CSF to 32.0 cm CSF (P =0.08). After 24 months the change in ICP demonstrated increasing effect size with a difference between the 2 arms of -8.2 cm CSF (P<0.001). Weight control was more effective in the BS arm compared to the CWM, with a difference of mean weight loss and excess weight loss of -18.3% (P< 0.001), and -46.4% (P<0.001), respectively. No difference was found between arms regarding perimetric mean deviation, HIT-6, or quality of life. The authors suggest that the negative secondary outcomes might have been influenced by the low number of patients recruited, which was expected considering the complex nature of the surgical intervention. Although more data is warranted to confirm the clinical benefits of BS for IIH treatment, this trial supports it as an effective treatment for patients with IIH and a BMI of 35 or higher.

## HEADACHE MANAGEMENT

Headache is the most common symptom and near-universal sequela of IIH. Despite the high morbidity, there is a lack of evidence to guide persistent headache management^14^. The treatments used for visual protection, either clinical with acetazolamide or surgical, have not shown benefit in improving headaches and therefore should not be prescribed for this purpose^18^. 

Management is mostly based on off-label treatments according to headache phenotype. IIH-related headaches can change phenotype throughout the disease, and a comprehensive clinical characterization is extremely important for phenotypic definition. A mixture of diverse phenotypes such as migraine, analgesic overuse headache, tension-type headache, and even CSF hypotension headache secondary to drainage procedures is not uncommon^33^. 

In almost 68% of IIH patients, the predominant phenotype is migraine, and therefore, prophylactic migraine therapy is commonly the treatment of choice. Attention should be given to avoid prophylactics that increase weight and worsen psychiatric comorbidities such as depression. Topiramate is the preferred oral prophylactic among neurologists. It reduces appetite, causes weight loss, and there is evidence that it promotes some degree of reduction in ICP. A limiting factor for topiramate is tolerability of the most reported adverse effects, like paresthesia and cognitive complaints. There are no randomized trials comparing the antimigraine therapeutics in IIH^33^.

Recently, a prospective open-label study evaluated patients with persistent headaches using Erenumab, a calcitonin gene-related peptide (CGRP) receptor monoclonal antibody. Erenumab significantly reduced (by 71%) days with moderate to severe headache and days with any headache (by 45%) from baseline to 12 months. In addition to improvement in pain, there was also a significant improvement in presenteeism, absenteeism, and analgesic use days. Erenumab had high tolerability without any patient leaving the study due to side effects. CGRP has been studied and implicated in the pathophysiology of migraine and may play a role in the pathophysiology of IIH-related headaches. Therefore, Anti-CGRP antibodies may be a potential therapeutic approach for IIH persistent headache^34^.
